# The Flatworm *Macrostomum lignano* Is a Powerful Model Organism for Ion Channel and Stem Cell Research

**DOI:** 10.1155/2012/167265

**Published:** 2012-09-11

**Authors:** Daniil Simanov, Imre Mellaart-Straver, Irina Sormacheva, Eugene Berezikov

**Affiliations:** ^1^Hubrecht Institute, KNAW, University Medical Center Utrecht, 3584 CT Utrecht, The Netherlands; ^2^Institute of Cytology and Genetics SB RAS, 630090 Novosibirsk, Russia; ^3^European Research Institute for the Biology of Ageing and University Medical Center Groningen, University of Groningen, 9713 AV Groningen, The Netherlands

## Abstract

Bioelectrical signals generated by ion channels play crucial roles in many cellular processes in both excitable and nonexcitable cells. Some ion channels are directly implemented in chemical signaling pathways, the others are involved in regulation of cytoplasmic or vesicular ion concentrations, pH, cell volume, and membrane potentials. Together with ion transporters and gap junction complexes, ion channels form steady-state voltage gradients across the cell membranes in nonexcitable cells. These membrane potentials are involved in regulation of such processes as migration guidance, cell proliferation, and body axis patterning during development and regeneration. While the importance of membrane potential in stem cell maintenance, proliferation, and differentiation is evident, the mechanisms of this bioelectric control of stem cell activity are still not well understood, and the role of specific ion channels in these processes remains unclear. Here we introduce the flatworm *Macrostomum lignano* as a versatile model organism for addressing these topics. We discuss biological and experimental properties of *M. lignano*, provide an overview of the recently developed experimental tools for this animal model, and demonstrate how manipulation of membrane potential influences regeneration in *M. lignano*.

## 1. Introduction

Ion channels represent a diverse family of pore-forming proteins. They are crucial for establishing voltage gradients across plasma membranes by allowing the flow of inorganic ions (such as Na^+^, K^+^, Ca^2+^, or Cl^−^) down their electrochemical gradients. Ionic flux through the channels provides the foundation for membrane excitability, which is essential for the proper functioning of neurons, cardiac, and muscle cells [1]. At the same time, ion channels serve many functions apart from electrical signal transduction. For example, Ca^2+^ is an important messenger, and changes in its intracellular concentrations influence numerous cellular processes in virtually all types of nonexcitable cells [2–4], including stem cells [5–7]. Besides, a number of ion channels are known to be directly involved in chemical signaling pathways in different cell types [8, 9]. As a result, mutations in genes encoding ion channel proteins have been associated with many disorders (so-called “channelopathies”), caused by dysfunction of both excitable (epilepsy, hypertension, cardiac arrhythmia) and nonexcitable (diabetes, osteopetrosis, and cystic fibrosis) cells [10]. Here we briefly describe the crucial role ion channels play in maintenance, proliferation, and differentiation of stem cells on the level of single cell and the whole organism. We discuss the importance of animal model systems, such as flatworms, for studying bioelectric signaling in complex morphogenesis during development and regeneration. Finally, we introduce the new flatworm model, *Macrostomum lignano*, and discuss its experimental potential for dissecting the roles of ion channels in stem cell regulation.

## 2. Ion Channels and Membrane Potential in Stem Cells

Numerous ion channels and pumps together with gap junction complexes form transmembrane voltage gradients. While quick changes of these membrane potentials (*V*_mem_) are best described in neurons, muscle, and cardiac cells, long-term steady-state *V*_mem_ levels are present in all other cells [11, 12]. Membrane potentials strongly correlate with the mitotic ability of different cell types, with the high resting potential associated with differentiated nondividing cells [13]. *V*_mem_ fluctuations during progression through the cell cycle have been reported in a number of cell types, and changes of membrane potential appear to be required for both G1/S and G2/S phase transitions [14–16]. Modulation of *V*_mem_ through applied electric fields or by inhibition of ion channels leads to cell cycle arrest in dividing cells [17–20], and artificial membrane hyperpolarization induces differentiation of mesenchymal stem cells [21]. On the other hand, electroporation (supposedly followed by membrane depolarization) activates cell hyperproliferation and de-differentiation [22].

**Figure 1:**
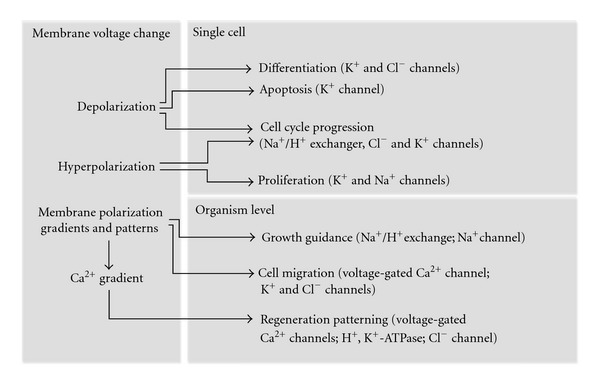
Ion channels and membrane voltage during regeneration. Changes of membrane potentials can directly affect different aspects of cell behavior and large-scale morphogenetic processes during regeneration. Ion channels and transporters implicated in these processes are mentioned in brackets.

On the level of multicellular organism, progression through the cell cycle should be strictly regulated and synchronized during such processes as development and regeneration in order to achieve a proper body patterning. Accordingly, stable and reproducible membrane polarization patterns have been recently described in various model organisms. Artificial modulation of these patterns during development or regeneration has a large impact on left-right asymmetry and anterior-posterior identity [23–27]. The role of bioelectric signaling in regeneration is comprehensively reviewed in [28] and schematically shown in Figure 1. Finally, modulations of membrane voltage have been observed in a large number of oncological disorders, and ion channels were proposed as cancer treatment targets [29, 30].

Thus, bioelectric signaling is an important mechanism of cell regulation, including stem cell maintenance, proliferation and differentiation. Recent findings suggest this control system to be well conserved in a wide range of animal phyla. However, the mechanisms linking membrane potential to the cell cycle, proliferation and differentiation, and the role of specific ion channels in this process remain largely unclear. The picture becomes even more complicated on the level of multicellular organism. Our understanding of the ways cells produce and receive bioelectric signals and translate them into positional information during development and regeneration is still fairly poor. While considerable knowledge about the role of membrane potential in stem cells was gathered recently from different species, the number of models used in this field is still limited. Expanding the range of model organisms used for functional studies of bioelectric signaling is crucial for better understanding of this control system and its role in complex morphogenesis.

## 3. Planarian Models in Ion Channel Research

Planarian flatworms are long-established models for stem cell and regeneration research. The adult stem cell system and regeneration capacity of the species *Planaria maculata* and *Planaria lugubris* were described by Morgan as early as in the end of 19th century [31, 32]. In our days the favorite planarian species for research in the regeneration field are *Schmidtea mediterranea* and *Dugesia japonica* [33, 34]. Planaria were also one of the first species in which stable membrane potential patterns were described, and their role in regeneration postulated. In 1940s and 1950s Marsh and Beams were able to specifically control establishing of anterior-posterior axis by providing bioelectrical signals to regenerating planaria fragments [35–37].

In the last 5 years considerable work was done in planaria on understanding the molecular and genetic mechanisms that allow cells to establish and maintain long-term membrane potential patterns and transduce bioelectric signals into proliferation and differentiation decisions. The importance of gap junction signaling in establishing anterior-posterior polarity during regeneration was shown [38], and the specific innexin gene, *Smedinx-11*, responsible for blastema (regenerating tissue) formation and stem cell maintenance identified [39].

The role of ion channels and pumps in the establishment of anterior-posterior axis during regeneration of planaria *D. japonica* was recently highlighted by groups of Michael Levin and Jonathan Marchant. *D. japonica*, which can regenerate an entire animal from a small part of a cut worm, has highly depolarized cell membranes in the head region, and highly polarized in the posterior part. In the cut worm this pattern is reestablished rapidly, regardless of the cutting plane [26]. After the wound is closed, blastema at all anterior-facing wounds gives origin to heads, while tails are regenerated from the posterior-facing wounds. The polarization pattern is altered by highly specific drugs against different ion channels and transporters, such as SCH-28080 (inhibitor of H^+^, K^+^-ATPase), ivermectin (IVM, activator of the invertebrate GluCl channels), or praziquantel (PZQ, activator of voltage-operated Ca^2+^-channels). Remarkably, induced depolarization itself is sufficient to drive ectopic anterior (head) regeneration even in posterior-facing blastemas, whereas membrane polarization of anterior-facing wounds blocks the head regeneration [25, 26]. The role of specific voltage-operated Ca^2+^ channels in regenerative patterning was addressed in the followup experiments [27].

Thus, planarian flatworms can be successfully used for ion channel and stem cell studies. Fascinating regeneration capacity of these animals, together with a wide range of research techniques established and optimized over the last 100 years, make planaria a very attractive model for studying bioelectric signaling during regenerative morphogenesis. However, due to inefficient sexual reproduction under laboratory conditions, classical genetic methods are not available in planarians, and reverse genetics methods are limited to RNA interference. Since genetic manipulation of these animals is difficult, no reproducible transgenesis methods are available for planaria [40].

## 4. Experimental Properties of the Flatworm *Macrostomum lignano*

**Figure 2:**
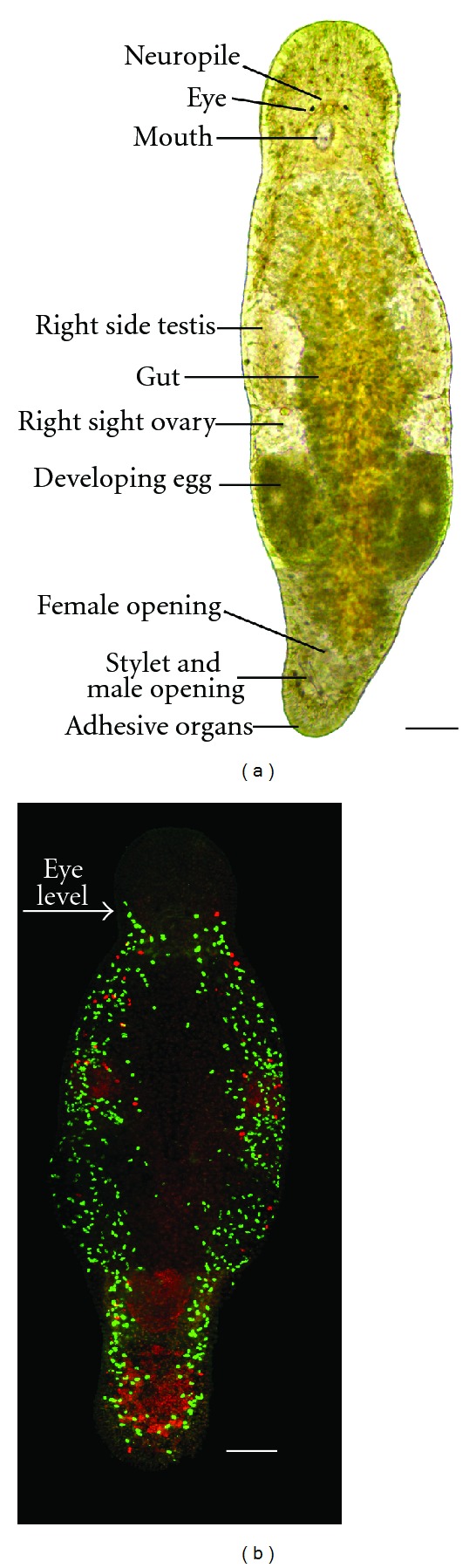
*Macrostomum lignano* as a model organism. (a) Bright field image of a living specimen. (b) Confocal projection of BrdU and phospho-histone H3 immunostaining after 30 minutes BrdU pulse in an adult worm (green: S-phase cells, red: mitotic cells). Scalebar 100 *μ*m.

During the last decade another flatworm, *Macrostomum lignano*, has emerged as a complementary model organism for regeneration research [41–44]. This marine free-living basal flatworm is about 1.5 mm long and consists of roughly 25000 cells. *M. lignano* is easy to culture in laboratory conditions, and populations of this animal are continuously maintained in the number of laboratories for over a decade. The generation time of the flatworm is short, with about two weeks of postembryonic development to sexually mature adult. Both juvenile and adult worms have clear morphology and are highly transparent (Figure 2(a)), greatly facilitating phenotyping and both fluorescent and non-fluorescent staining. The regeneration capacity of *M. lignano *is provided by roughly 1600 neoblasts (adult stem cells) located mesodermally. Proliferation activity of these cells can be easily studied using BrdU labeling, performed by simple soaking [43, 45] (Figure 2(b)). Importantly, *M. lignano* is nonself fertilizing hermaphrodite and has exclusively sexual reproduction. Well-fed adult animals generate a lot of embryos all year through (one animal lies on average one egg a day), making it accessible for genetic manipulation. *In situ* hybridization [43] and RNA interference (by soaking) [45] protocols are established and optimized for *M. lignano*, and a number of tissue-specific monoclonal antibodies are available [41]. Basic culturing and experimental properties of *M. lignano* are summarized in Table 1.

Considerable progress has been made in the past three years towards establishing *M. lignano* as versatile stem cell research model for the genomics era. The work on *M. lignano* genome assembly and annotation is in progress (Berezikov and colleagues), and draft genome and transcriptome assemblies are publicly available at http://www.macgenome.org/. Comparing transcriptome data obtained from irradiated (neoblast-depleted) and control worms provided the insight into the role of a number of genes in regeneration, while stage-specific transcriptome data showed the temporal expression of *Macrostomum* genes through development (Simanov et al., in preparation). Most importantly, proof-of-principle for transgenesis in *M. lignano* has been demonstrated and first stable transgenic GFP-expressing lines of *M. lignano* have been established (Demircan, De Mulder, Berezikov et al., in preparation). Thus, biological and experimental properties of *M. lignano*, combined with its rapidly expanding experimental toolbox, make this animal an attractive and powerful model organism for stem cell and regeneration research. Its astonishing ability to resist *γ*-irradiation and recover after being exposed to it makes the neoblast system of this animal exceptional even for flatworms [46]. Moreover, fascinating but yet poorly understood link between regeneration and rejuvenation provides an exciting opportunity of using *M. lignano* as a model for ageing research [47].

## 5. Ion Channels and Regeneration in *M. lignano*

Unlike planarian flatworm species, *M. lignano *is unable to regenerate the head under normal circumstances. Posterior-facing blastemas give origin to fully functioning tails with all its organs and structures, whereas anterior-facing wounds develop blastema layer but the actual regeneration can only happen if the worm was amputated in front of the brain (at the very tip of the head). Thus, anterior fragments of the worm, having a functional head, can regenerate the whole body in 2-3 weeks, while posterior fragments normally die 5–10 days after losing the head [42]. These differences in the head regeneration capacity between *M. lignano* and planarians, and the ability to induce ectopic head regeneration in *D. japonica* by the manipulation of membrane voltage gradients, prompted us to investigate how these findings in planarians translate into *M. lignano.*

**Figure 3:**
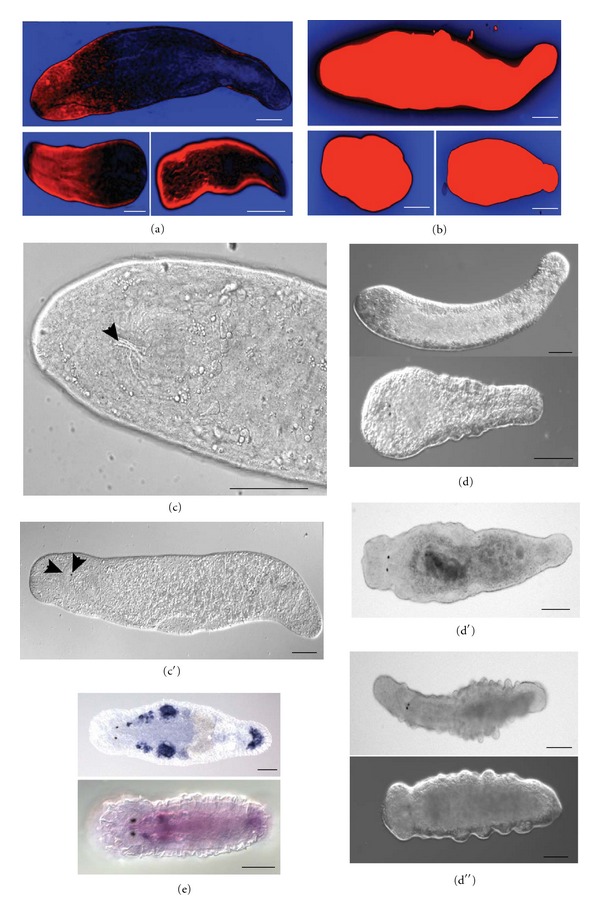
Bioelectric signaling and stem cells in *M. lignano*. (a-b) DiBAC_4_(3) staining of intact worm (top), anterior (left bottom) and posterior (right bottom) fragments. (a) control worm, (b) worm treated with 1 *μ*M IVM. Blue is more polarized than black, black is more polarized than red. (c-c′) Regeneration of head-specific structures after 1 *μ*M IVM treatment. Arrowheads in (c) indicate regenerated pharynx, in (c′) regenerated eye and half of the brain. (d-d′′) intact worms exposed to high doses of IVM (2 *μ*M in d and 4 *μ*M in d′) and PZQ (150 *μ*M in d′′). (d) head regression; (d′) square head; (d′′) bulges and outgrowth. (e) *In situ* hybridization results in adult (top) and juvenile (bottom) animals with the probe against RNA815_5834 transcript from ML110815 transcriptome assembly (voltage-gated sodium channel). In juvenile worm this gene is expressed almost ubiquitously, and in adults expression is only detected in gonads and (likely) in somatic stem cells. Strong signal in the adhesive glands in the tail is likely a common artifact.

DiBAC_4_(3) voltage-reporting dye stainings (as described in [48]) showed that membrane voltage pattern in *M. lignano *is similar to the one observed in *D. japonica *[26]—the anterior part is highly depolarized, while the tail is relatively polarized. In the cut worms this pattern is quickly reestablished in the anterior head-containing fragments, while the posterior headless fragments do not show any clear anterior-posterior polarization gradient and do not regenerate (Figure 3(a)). Just like in planarians, membrane polarization patterns in *M. lignano* can be altered using drugs against ion channels. IVM induces depolarization of the membranes of intact and cut worms, both in anterior and posterior regions (Figure 3(b)). Posterior-facing blastemas still regenerate the tails after treatment, though the full regeneration takes longer than normally. Anterior-facing wounds treated with IVM develop blastema, and some tissue growth is often observed within a week after wound closure. IVM-treated headless fragments always move more actively and survive longer comparing to control fragments. Strikingly, 1.5% of posterior fragments after IVM treatment are able to regenerate head-specific structures and, in a few cases, a fully functional head (Figures 3(c) and 3(c′)). PZQ causes the same depolarization effect but does not have any effect on regeneration patterning at tested concentrations (data not shown). Intact animals exposed to high doses of IVM or PZQ display phenotypes that in planarian flatworms are stereotypically associated with stem cell loss or disorder [49–52]. *M. lignano* animals treated with 2 *μ*M IVM gradually lose anterior identity, with no head-specific structures left 7–9 days after treatment (Figure 3(d)). After exposure to higher doses of IVM (3-4 *μ*M), worms develop characteristic square head due to partial tissue loss in the most anterior part of the body, get paralyzed and die 3-4 days after treatment (Figure 3(d′)). High concentration of PZQ in culturing media causes formation of bulges, mainly in the posterior part of the body (Figure 3(d′′)). This phenotype is completely different from the one observed after IVM treatment, suggesting specific action of the drugs.

These pilot experimental results show that *M. lignano* can be successfully used as a model for ion channel and stem cell studies. The complete transcriptome and established *in situ *hybridization and RNA interference methods, in combination with chemical treatment make it possible to address the function of specific ion channels in development, tissue turnover, and regeneration. For example, comparison of transcriptome data from irradiated (stem cell-deficient) and nonirradiated animals (Simanov et al., in preparation) highlights a number of ion channel genes expressed specifically in dividing cells (Figure 3(e)), and future elaborated studies of such genes may provide novel insights into the role of bioelectric signaling in stem cell maintenance and differentiation. Importantly, a significant number of ion channels are well-conserved between *M. lignano *and human (Table 2), increasing the relevance of findings in flatworms to understanding ion channels and stem cells in human situation.

## 6. Future Directions

We advocate that *Macrostomum lignano *has great potential as a model for ion channel and stem cell research. The genetic toolbox available for this organism is already useful enough to address a wide range of scientific problems, and more methods and approaches will be optimized and used in this flatworm in the near future.* M. lignano *is a small animal and it is cultured in water, which makes it easy to apply different chemicals to the worms. Another major advantage of the animal is its high transparency. Phenotypic changes, fluorescent signals or certain transgene expression can be observed in any part of the body, as well is on the whole organism scale. For example, various fluorescent reporter dyes can be just added to culturing media in order to enable real-time *in vivo* monitoring of membrane potentials, pH, and ion flows [53]. Short generation time and efficient reproduction of *M. lignano* make logistics of large-scale experiments, such as drug screens, feasible in this animal.

**Figure 4:**
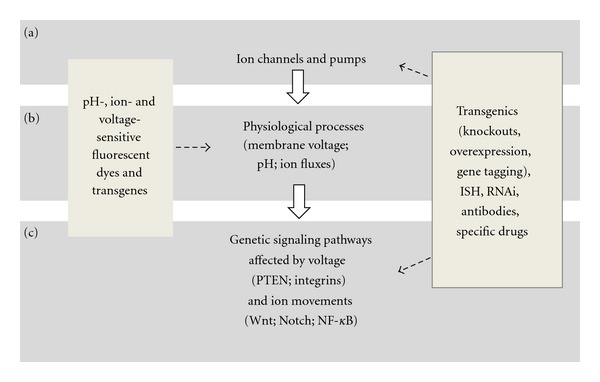
Approaches to study the roles of ion channels in regulation of stem cells in *M. lignano.* (a) Expression, localization, and function of ion channels and pumps that give rise to bioelectric signals can be addressed in *M. lignano* by established methods such as RNAi or *in situ* hybridization (ISH) in combination with specific drugs, antibodies, and transgenics. (b) Changes in ion flows, pH and membrane voltage caused by these channels and pumps can be detected with sensitive fluorescent dyes or followed *in vivo* in mutants expressing pH- or ion-sensitive forms of fluorescent proteins. (c) These processes affect known (and possibly unknown) genetic signaling pathways via different mechanisms including changes of Ca^2+^ concentrations, voltage-sensing domains of proteins, and voltage-gated transport of signaling molecules. These pathways and functional links between genetic and epigenetic mechanisms of stem cell function regulation can be studied in transgenic mutant lines with the help of RNAi and ISH techniques.

As a model, *M. lignano* offers an exciting opportunity to bridge the gap between bioelectric signaling and genetic pathways involved in stem cell functions. The expression pattern and function of any gene can be determined by *in situ* hybridization and RNAi protocols, but it is transgenics that can bring such studies to the whole new level. Transgenic reporter lines expressing pH-sensitive or Ca^2+^-sensitive fluorescent proteins [54, 55] would make a perfect tool to visualize bioelectric phenotypes during drug- or RNAi-screens. Overexpression of ion channels or even certain subunits would help to better understand their functions and interactions. Targeted genome editing by Zinc Finger Nucleases have not been tested yet in this animal but should be also feasible and potentially can be used to generate ion channel knockout and knock-in lines [56, 57]. The same method allows fluorescent tagging of genes of interest and analysis of their expression, localization, and functions at the endogenous level [58]. Sexual reproduction and lack of self-fertilization make possible crossing different lines of *M. lignano* and hence to use the power of classical genetics approaches in this animal. Taken all together, we are convinced that *M. lignano* is poised to become a productive model to study relations between ion channels and stem cell regulation (Figure 4).

## Figures and Tables

**Table 1:** Biological and experimental properties of *M. lignano*.

Size	1 mm
Total cell number	±25.000
Neoblasts	±1600
Transparency	Highly transparent
Culturing media	f/2 (sea water based)
Feeding	Diatom algae (*Nitzschia curvilineata*)
Embryogenesis	5 days
Generation time	18 days
Nervous, muscle system, and gonads	Simple
Stem cell system	Pluripotent
BrdU/H3 staining	Yes (easy by soaking)
RNA interference	Yes (easy by soaking)
Accessibility to eggs	Single eggs (one egg/day per animal)
Transgenics	Possible, by injection into eggs

**Table 2:** Major categories of ion channel genes conserved between *H. sapiens* and *M. lignano. *

GO term	Description	*H*	*M*	Human genes
GO:0004889	Acetylcholine-activated cation-selective channel activity	13	132	CHRNA4, CHRNE, CHRNA10, CHRNB1, CHRNB3, CHRNA6, CHRNA3, CHRND, CHRNB2, CHRNB4, CHRNA9, CHRNA2, CHRNA7
GO:0004931	Extracellular ATP-gated cation channel activity	5	15	P2RX6, P2RX7, P2RX5, P2RX4, P2RX2
GO:0004970	Ionotropic glutamate receptor activity	12	67	GRIN1, GRIA4, GRIN2A, GRIK2, GRIK1, GRIA1, GRIK4, GRIA2, GRIK3, GRID1, GRIN3A, GRIK5
GO:0005216	Ion channel activity	6	23	PKD1L2, MCOLN3, MCOLN2, PKD2L2, PKD2L1, PKDREJ
GO:0005221	Intracellular cyclic nucleotide activated cation channel activity	2	5	KCNA10, CNGA3
GO:0005222	Intracellular cAMP activated cation channel activity	1	2	HCN4
GO:0005223	Intracellular cGMP activated cation channel activity	1	1	CNGB3
GO:0005229	Intracellular calcium activated chloride channel activity	2	3	ANO1, ANO2
GO:0005232	Serotonin-activated cation-selective channel activity	2	3	HTR3B, HTR3A
GO:0005237	Inhibitory extracellular ligand-gated ion channel activity	2	3	GABRA6, GABRB2
GO:0005242	Inward rectifier potassium channel activity	6	26	KCNH6, KCNJ12, KCNK6, KCNJ8, KCNQ5, KCNH7
GO:0005245	Voltage-gated calcium channel activity	8	27	CACNA1C, CATSPER1, CACNG7, CACNG5, CACNB1, CACNA1B, CACNB2, CACNA1E
GO:0005247	Voltage-gated chloride channel activity	6	14	CLCN7, CLCN4, CLIC1, CLIC4, CLIC6, CLCN3
GO:0005248	Voltage-gated sodium channel activity	8	19	SCN3A, SCN2A, SCN4A, PKD2, SCN8A, SCN5A, SCN9A, SCN11A
GO:0005249	Voltage-gated potassium channel activity	23	75	KCTD12, KCTD21, KCNH3, KCTD10, KCTD3, KCTD6, KCNAB3, KCTD2, KCTD15, KCTD7, KCNH4, KCNB1, KCTD9, KCNH8, KCNC3, KCNC2, KCTD16, KCND1, KCNC1, KCNV2, KCNH5, KCTD1, KCTD20
GO:0005250	A-type (transient outward) potassium channel activity	3	11	KCNIP2, KCND3, KCND2
GO:0005251	Delayed rectifier potassium channel activity	8	26	KCNA3, KCNB2, KCNH2, KCNA1, KCNA5, KCNQ1, KCNA2, KCNH1
GO:0005254	Chloride channel activity	17	55	CLCA1, ANO3, GABRB3, GABRA2, GABRB1, ANO7, ANO9, ANO4, GABRG2, CLCA4, CLCC1, ANO6, GABRQ, GABRG1, ANO10, GABRA4, GABRG3
GO:0005261	Cation channel activity	7	33	TRPM3, TRPV4, TRPM6, TRPC7, TMEM38A, TRPV1, HCN2
GO:0005262	Calcium channel activity	7	51	TRPM1, TRPM7, TRPM8, TRPV5, TRPM5, TRPM4, TRPV6
GO:0005267	Potassium channel activity	13	27	KCNC4, KCNK16, KCNK10, KCNG1, KCNK2, KCNK5, KCNK3, KCNK12, KCNQ4, KCNK17, KCNIP1, KCNIP4, KCNK9
GO:0005272	Sodium channel activity	4	40	HCN1, NALCN, ACCN4, TRPM2
GO:0008308	Voltage-gated anion channel activity	2	3	VDAC1, VDAC2
GO:0008331	High voltage-gated calcium channel activity	7	38	CACNA1A, CACNA2D4, CACNA1D, CACNA2D1, CACNA1S, CACNA2D3, CACNA2D2
GO:0008332	Low voltage-gated calcium channel activity	3	18	CACNA1H, CACNA1I, CACNA1G
GO:0015269	Calcium-activated potassium channel activity	9	49	KCNMA1, KCNN1, KCNT2, KCNN2, KCNT1, KCNU1, KCNMB2, KCNK18, KCNN3
GO:0015276	Ligand-gated ion channel activity	2	4	CLCA2, CNGB1
GO:0015279	Store-operated calcium channel activity	5	40	TRPC4, TRPC6, ORAI1, TRPA1, TRPC3
GO:0015280	Ligand-gated sodium channel activity	8	97	SCNN1B, SCNN1G, ACCN1, ACCN3, SCNN1A, ACCN5, ACCN2, SCNN1D
GO:0022824	Transmitter-gated ion channel activity	4	34	GLRA2, GLRA4, GLRA1, GLRA3
GO:0030171	Voltage-gated proton channel activity	1	3	HVCN1
GO:0072345	NAADP-sensitive calcium-release channel activity	2	3	TPCN1, TPCN2

	Total	199	947	
	Total number of genes in these GO categories	390		

*H*: number of different ion channel genes in human with homologs in *M. lignano. M*: number of transcripts in *M. lignano de novo* transcriptome assembly ML110815 with homology to ion channel genes in human. Note that alternatively spliced transcripts are counted separately in the *M. lignano* transcriptome assembly, hence the total reported number of transcripts is higher than the number of corresponding human genes. For this classification, genes were assigned to the least frequent available GO term within predefined list of ion channel-related GO terms (molecular function domain).
